# P-2060. Effectiveness of BNT162b2 COVID-19 Vaccination Against Long COVID Among Older Adults: A Nationwide Study

**DOI:** 10.1093/ofid/ofae631.2216

**Published:** 2025-01-29

**Authors:** Manuela Di Fusco, Abby Rudolph, Laura L Lupton, Joseph C Cappelleri, Alon Yehoshua, Laura A Puzniak, Santiago M C Lopez, Xiaowu Sun

**Affiliations:** Pfizer Inc, New York, New York; Pfizer Inc, New York, New York; CVS Health Clinical Trial Services, Woonsocket, Rhode Island; Pfizer Inc., Groton, Connecticut; Pfizer Inc., Groton, Connecticut; Pfizer Inc., Groton, Connecticut; Pfizer Inc, New York, New York; CVS Health, Woonsocket, Rhode Island

## Abstract

**Background:**

Long COVID includes new or persisting symptoms/signs/conditions impacting >1 organ/system following acute infection. The clinical presentation varies across patients and estimates of burden depend on the definition. Evidence on effectiveness of BNT162b2 BA.4/5 bivalent COVID-19 Vaccine (BNT162b2 bivalent) against long COVID remains scarce. This study assessed vaccine effectiveness (VE) of BNT162b2 bivalent for preventing long COVID symptoms among older adults.

**Table 1.**
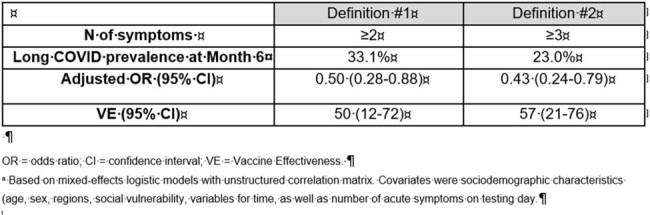
Long COVID prevalence and vaccine effectiveness of BNT162b2 a against long COVID symptoms among older adults ≥50 years old

**Methods:**

Symptomatic US adults ≥50 years old testing positive for SARS-CoV-2 via RT-PCR and rapid antigen at CVS Health were recruited between 03/02-05/18/2023 (CT.gov: NCT05160636). Participants reported presence or absence of each of 30 long COVID symptoms at 4 weeks, months 3 and 6 after testing. The CDC long COVID definition (new-onset or symptoms persisting ≥4 weeks after acute infection) was operationalized as the presence of ≥2 or ≥3 new or persistent symptoms consistent with long COVID from week 4 to month 6. The odds ratio (OR) of long COVID was computed for those who had received vs. had not received the BNT162b2 bivalent dose (vaccinated versus unvaccinated) before infection using mixed-effects logistic models, adjusting for multiple covariates. Absolute VE versus unvaccinated was calculated as (1‒OR)x100.

**Results:**

Of 277 older adults aged ≥50 years recruited in the study, 172 received BNT162b2 and 105 did not. Mean time since last bivalent dose was 168 days. All study participants reported ≥1 long COVID symptoms through month 6. The prevalence of ≥2 and ≥3 long COVID symptoms at month 6 was 33.1% and 23%, respectively. Those who received the BNT162b2 bivalent dose were less likely than those who didn’t to report ≥2 symptoms (27.0% vs 44.4%; adjusted OR 0.50, 95% CI 0.28-0.88) and ≥3 symptoms (16.5% vs 34.9%; adjusted OR 0.43, 95% CI 0.24-0.79) from 4 weeks to 6 months since testing positive. Absolute VE ranged 50-57% (Table 1).

**Conclusion:**

Despite high levels of immunity, especially among older adults, the post-pandemic burden of long COVID remains high, but is significantly lower for those vaccinated. Between 4 weeks and 6 months after testing positive, older adults vaccinated with BNT162b2 bivalent had approximately half the odds of long COVID than those unvaccinated.

**Disclosures:**

Manuela Di Fusco, PhD, Pfizer Inc.: Employee|Pfizer Inc.: Stocks/Bonds (Public Company) Abby Rudolph, PhD, Pfizer Inc: Employer|Pfizer Inc: Stocks/Bonds (Public Company) Laura L. Lupton, MD, MHSA, CVS Health: Employee|CVS Health: Stocks/Bonds (Public Company) Joseph C. Cappelleri, PhD, Pfizer Inc.: Employee|Pfizer Inc.: Stocks/Bonds (Public Company) Alon Yehoshua, PharmD, MS, Pfizer Inc: Employer|Pfizer Inc: Stocks/Bonds (Public Company) Laura A. Puzniak, PhD. MPH, Pfizer Inc.: Employee|Pfizer Inc.: Stocks/Bonds (Public Company) Santiago M.C. Lopez, MD, Pfizer Inc.: Employee|Pfizer Inc.: Stocks/Bonds (Public Company) Xiaowu Sun, PhD, CVS Health: Employee|CVS Health: Stocks/Bonds (Public Company)

